# New Appliances and Things Medical

**Published:** 1894-07-07

**Authors:** 


### NEW APPLIANCES AND THINGS MEDICAL.

### MIGRANIN.

(Burroughs, Wellcome, and Co., Snow Hill, E.C.)

Amongst a host of new synthetical preparations, the outcome of the ever active energy of the German laboratories, our attention has been called by Messrs. Burroughs and Wellcome to the new drug, migranin. It is, perhaps, hardly fair to call this a new drug, as it has been on trial for some years in Germany, but as far as the profession in England is concerned it may certainly de described as a new preparation. Chemically, migranin may be regarded as citrate of antipyrin-caffein, as it is a combination of Knoll's antipyrin with citric acid and the addition of caffein, each ingredient being added in definite proportions. Whether, however, migranin is to be considered as a compound or simply a mixture of the above constituents does not appear to have been definitely settled. Clinically, however, this is an unimportant point since its therapeutic action is the question with which we are chiefly interested. It is claimed that this drug is a specific for cases of migranin, and in the hands of Dr. Martin Overlach it has met with such signal success that in all cases of this intractable disorder it should be certainly given a trial. Antipyrin and caffein have each separately so often given relief from this condition that it might safely be predicted that a correct combination of the two drugs might have a twofold beneficial effect, and this certainly seems to be the case with migranin.

### NEW SICK-ROOM CHARTS.

(Stickland and Co., Pharmaceutical Chemists, South Kensington.)

These charts have several new features and certainly are an improvement in many ways on the older forms. Each complete chart' is composed of eight folios, neatly bound and fixed to a cardboard mount, which may be hung on the wall or on the back of the bed. The first seven leaves are devoted to a record of nutriment, stimulant, and medicine, for the guidance of the nurse, and for the assistance of the physician there are blank columns to be filled in by the nurse, containing notes as to duration of sleep, action of the bowels, quantity of urine passed, pulse rate, respiration, &c. Each chart is a complete one for the 24 hours, divided up into half-hourly intervals. The eighth chart is a temperature chart for the seven days. The clinical history of the patient for the twenty-four hours may thus be read by the medical attendant at a glance, instead of having to refer to two or three separate pages, as is usually the case. The cheap rate at which these charts are supplied to the profession is an additional point in their favour.

